# Identification of multidrug-resistant enterobacteriaceae in fecal samples from infants residing in Talara, Piura, Peru

**DOI:** 10.17843/rpmesp.2022.394.11870

**Published:** 2022-12-09

**Authors:** Arturo Octavio Gonzales-Rodríguez, Javier Ignacio Castillo Horna, Edgar Gonzales Escalante

**Affiliations:** 1 Faculty of Human Medicine, Universidad de Piura, Lima, Peru Universidad de Piura Faculty of Human Medicine Universidad de Piura Lima Peru; 2 Faculty of Pharmacy and Biochemistry, Instituto de Investigaciones en Bacteriología y Virología Molecular (IBaViM), Universidad de Buenos Aires, Argentina Universidad de Buenos Aires Faculty of Pharmacy and Biochemistry Instituto de Investigaciones en Bacteriología y Virología Molecular (IBaViM) Argentina

**Keywords:** Newborn, *Escherichia coli*, Drug Resistance, beta-Lactamases, Quinolones, Peru, Coliforms

## Abstract

Fecal colonization by antimicrobial-resistant bacteria in infants is a potential risk for future antibiotic therapy. We aimed to determine the sociodemographic characteristics and frequency of infants who were fecal carriers of ciprofloxacin-resistant enterobacteriaceae (FCCRE) and their associated resistance genes. We analyzed fecal samples from 41 infants from the district of Talara, Piura, Peru in 2019. The presence of 3 quinolone resistance genes was evaluated: *aac(6’)-Ib-cr, qnrB* and *oqxA* as well as of 2 beta-lactamase genes: *bla*_CTX-M_,*bla*_PER-2_. We found that 68% of infants were FCCRE, *Escherichia coli* (83.3%) was the most frequent bacteria. The genotypic analysis detected: *oqxA* (41.1%), *qnrB* (26.7%), *aac(6’)-Ib-cr* (20%) and the *bla*_CTX-M_ gene (93.3%) of the isolates with beta-lactamases. The high frequency of FCCRE alerts us of the potential risk of this antibiotic family becoming less useful over time.

## INTRODUCTION

Antimicrobial resistance (AMR) is a serious public health problem. The World Health Organization (WHO) has estimated that by 2050 all antibiotics will be ineffective. The rapid, early, and widespread dissemination of AMR genes is considered the main reason for this ^1^. Colonization with drug-resistant bacteria is a major health risk due to the potential transfer of resistance genes to pathogenic bacteria and their easy dissemination between individuals. The intestinal microbiota has been reported to be a major source of urinary tract, respiratory, and bloodstream infection ^2^. 

Quinolones are a broad family of antibiotics whose resistance increased rapidly in the 1990s ^3^. Mutations in the quinolone resistance determinant region (QRDR) are the main mechanism that confers a high level of resistance. Plasmid-mediated quinolone resistance markers (PMQR), such as Qnr proteins, the AAC(6’)-Ib-cr enzyme and the OqxAB pump, exert a low level of resistance. However, they play an important role in the selection of chromosomal mutants in QRDR ^3^. 

ß-lactams are the main family of antibiotics, and extended-spectrum ß-lactamases (ESBL) represent the main mechanism of resistance to these antibiotics ^4^. CTX-M enzymes, predominant worldwide, are made up of different groups: CTX-M-group 1, CTX-M-group 2, CTX-M-group 8, CTX-M-group 9, CTX-M-group 25 and KLUC group. Groups 1 and 2 are the most widespread in Latin America ^4^.

In addition to being related to the use and abuse of antimicrobials, AMR is also related, although not directly, to the socioeconomic status of the family and the economic and healthcare development of a country ^5^. The study of populations potentially protected from colonization by AMR bacteria offers an opportunity to gather necessary evidence on the microbial quality of the environment. Infants are such a population, due to their limited motor capacity, low exposure to antimicrobials and limited nutritional variety ^6^.

This study aimed to determine the frequency and sociodemographic characteristics of infants who are fecal carriers of ciprofloxacin-resistant enterobacteriaceae (FCCRE) and the presence of PMQR and ESBL genes in the district of Talara, Piura region, Peru.

**KEY MESSAGES**

Motivation for the study: infants, due to restricted nutritional intake, limited motor capacity and low antibiotic exposure, are a population protected from multidrug-resistant bacteria.

Main findings: in this study, 68% of the infants were colonized by quinolone-resistant bacteria, mainly by E. coli (83.3%). The gene mainly associated with quinolone resistance was oqxA (41.4%). Also, half of the isolates were ESBL producers; and resistance was caused by the blaCTX-M gene in 93.3% of the isolates.

Implications: these findings alert us about the high presence of antimicrobial-resistant bacteria in a vulnerable population; which shows the potential risk of this antibiotic family becoming less useful over time.

## THE STUDY

A descriptive cross-sectional study was carried out. We enrolled 41 infants between 3 and 12 months of age from the district of Talara, Piura region, Peru. Of these, 28 infants were enrolled from the Talara Baja area based on information provided by the healthcare center and 13 infants from the Talara Alta area by identifying infants in the community who met the selection criteria.

Samples were collected between September and December 2019. The sample size was not calculated for this study. Sample selection was non-probabilistic by convenience under the following selection criteria: having informed consent from the mother for the child’s participation, having provided a stool sample, having a frequency of breastfeeding greater than four times per day, and having been born vaginally. We excluded infants whose mothers had consumed antibiotics 15 days prior to stool sample collection.

### Search and identification of quinolone-resistant enterobacteria

Stool samples were transported in Cary-Blair medium to the Microbiology and Immunology Laboratory (LMI) of the University of Piura in Lima for processing. Stool samples were placed on MacConkey agar supplemented with 2 mg/L ciprofloxacin for presumptive isolation of quinolone-resistant enterobacteria.

Identification and antimicrobial sensitivity profiling was carried out with the Vitek 2 compact automated system (Biomeriux, France); the interpretation process followed the Clinical and Laboratory Standards Institute (CLSI) recommendations ^7^. Phenotypic detection of ESBL was carried out by the double-disk method according to CLSI recommendations ^7^.

### Detection of quinolone and beta-lactam resistance genes.

Bacterial DNA was extracted using DNA Purification kit GeneJetGenomic (ThermoScientific), following the manufacturer’s recommendations. The presence of 3 PMQR genes: *aac(6’)-Ib-cr, qnrB*, and *oqxA* was determined using primers previously described in the literature ^8^. In addition, the presence of two ESBL-associated resistance genes* bla*_CTX-M _and *bla*_PER-2_ was studied among the isolates with evidence of ESBL ^8^. We searched for CTX-M groups 1, 2 and 9 in the isolates in which the* bla*_CTX-M_ gene was detected ^8^.

### Clonal relationship

The clonal relationship between ESBL-producing isolates was determined using the ERIC-PCR technique, as described in the literature ^9^. The Past software version 4.0 was used to integrate, in a dendogram, the results through the UPGMA algorithm. Isolates showing more than 90% identity were considered clonally related.

### Survey

A structured questionnaire was administered to 37 mothers, 11 with non- FCCRE children and 26 with PFRC children (the questionnaire was not administered to 2 mothers with FCCRE children and 2 mothers with ciprofloxacin-sensitive children). The questionnaire was based on the questions in Chapter 1 “Household and population characteristics”, module “Characteristics of dwellings and households” of the Demographic and Family Health Survey (ENDES) - 2014 ^10^. In addition, information was collected on the age and sex of infants.

### Statistical analysis

SPSS Statistics for Windows, Version 25.0. (Armonk, NY: IBM Corp) was used for the statistical analysis. Qualitative variables were described using frequency graphs; and quantitative variables were described using tables. Fisher’s exact test was applied to evaluate differences between groups carrying quinolone-resistant and quinolone-sensitive enterobacteria and the Student’s t-test was used to compare means. P values <0.05 were considered significant. 

### Ethical considerations

Ethical approval for the main study “Association of postnatal stress with the quality of the breast milk microbiome and its relationship with iron deficiency anemia in infants” (Project code: PI2008-UDEP) was granted by the Institutional Research Ethics Committee of the Faculty of Human Medicine of the San Martín de Porras University. Informed consent and assent were obtained from the mothers of each infant prior to enrollment.

## FINDINGS

Forty-one infants were enrolled, with an average age of 7.5 months; 48.6% were male, 51.4% were female, and 68.3% (28/41) were FCCRE.

*Bacterial identification and antibiotic susceptibility testing*

**Figure 1:**
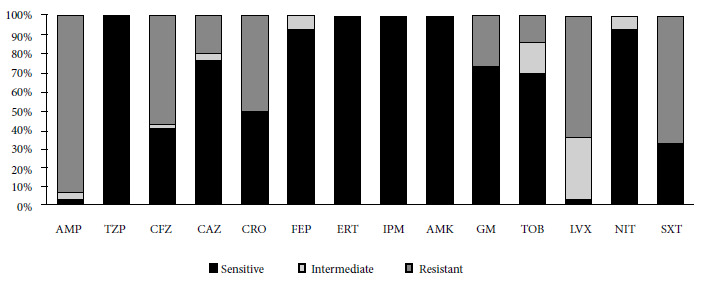
Antimicrobial susceptibility profile (n =30). AMP: ampicillin; TZP: piperacillin/tazobactam; CFZ: cefzazoline; CAZ: ceftazidime; CRO: ceftriaxone; FEP: cefepime; ERT: ertapenem; IPM: imipenem; AMK: amikacin; GM: gentamicine; TOB: trobamycin; LVX: levofloxacin; NIT: nitrofurantoin; and SXT: trimethoprim/sulfamethoxazole

Thirty isolates resistant to ciprofloxacin were recovered from 28 infants (two types of bacteria were recovered from each of two infants). The species isolated were *Escherichia coli* (25/30), *Citrobacter freundii* (2/30), *Enterobacter cloacae* subsp *cloacae* (1/30), *Klebsiella pneumoniae* subsp *pneumoniae *(1/30) and *Hafnia paralvei* (1/30). The antimicrobial susceptibility profile is shown in Figure 1, highlighting the levels of resistance to ampicillin (AMP) and trimethoprim/sulfamethoxazole (SXT), 93.3% and 66.7%, respectively. In addition, ESBL was detected in 15 of 30 isolates after the phenotypic evaluation.

*Genotypic detection and clonality profiling*

**Figure 2:**
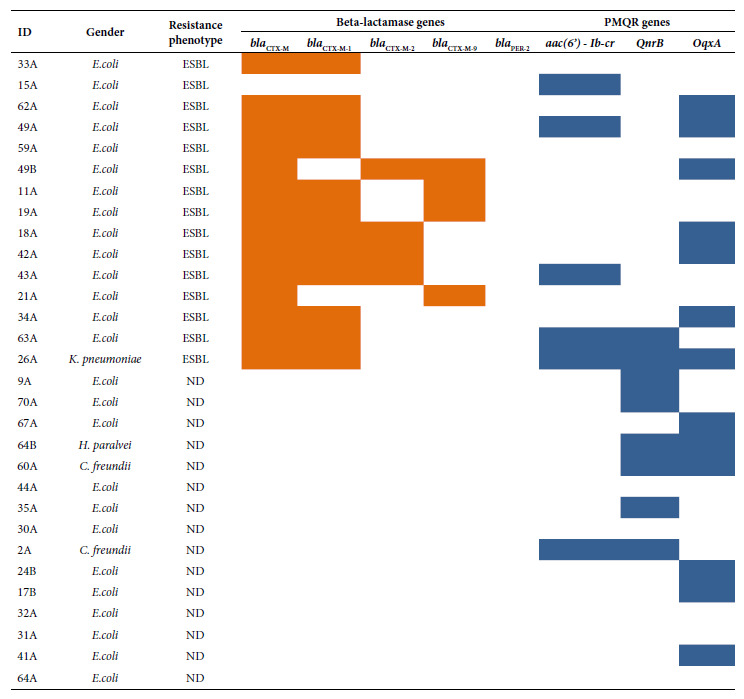
Distribution of ESBL and PMQR type resistance genes according to phenotype and bacterial genus. , : Presence; :absence.

**Figure 3:**
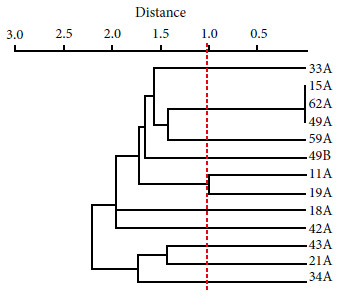
Dendrogram based on Euclidean genetic distance using the unpaired method of analysis at the arithmetic mean (UPGMA), constructed by presence-absence analysis of consensus intergenic regions by PCR (ERIC-PCR), for ESBL E. coli isolates.

The following PMQR genes were detected: *oqxA* (13/30), *qnrB* (8/30) and *aac*(6’)-*Ib-cr* (6/30). The *bla*_CTX-M_ gene was detected (14/15) in ESBL producers. Strains with the *bla*_CTX-M_ gene belonged to the following groups: *bla*_CTX-M-group 1_ (12/14), *bla*_CTX-M-group 2_ (4/14) and *bla*_CTX-M-group 9_ (4/14). The presence of the *bla*_PER-2_ gene was not detected (Figure 2). The study of the phylogenetic relationship between ESBL-producing *E. coli *isolates revealed up to 10 clonal groups; the related isolates were: 15A, 62A and 49A (Figure 3).

*Demographic and economic analysis*

No statistical difference was found between the infants who were fecal carriers of ciprofloxacin-sensitive and resistant enterobacteria with regard to sex. However, a statistical difference was found according to age (p = 0.007). Table 1 shows the results for other household variables. Finally, no statistical difference was found between the economic status and any of the evaluated variables.

**Table 1:** Sociodemographic analysis between infants who are fecal carriers of ciprofloxacin-resistant enterobacteriaceae (FCCRE) and non-FCCRE infants.

Variables	non-FCCRE infants (n =11) *	FCCRE infants (n = 26) *	p-value
Age (months)- mean (SD)	6.27 (2.49)	8.64 (2.18)	0.007^b^
Sex			
Men	6	12	0.721^a^
Women	5	14	
Type of housing			
Independent house	8	14	0.609^a^
Apartment in building	0	1	
House in a “quinta”	0	1	
Dwelling in a tenement house (alley, lot, or yard)	0	0	
Hut or cabin	1	7	
Improvised household	2	3	
Housing floor material			
Parquet or polished wood	1	1	0.862^a^
Asphalt, vinyl, or similar sheeting	0	2	
Tiles, terrazzo or similar	2	4	
Cement	7	17	
Soil	1	2	
No. rooms/No. sleeping rooms, mean (SD)	1.01 (0.77)	1.10 (1.54)	0.813^b^
Housing condition			
Rented	0	4	0.538^a^
Own, fully paid for	5	8	
Own, by trespassing	3	7	
Other	3	7	
Access to water			
Public network, inside the house	9	18	0.863^a^
Public network, outside the dwelling, but inside the building	0	0	
Public fountain	1	2	
Tanker truck or similar	1	2	
Well (groundwater)	0	0	
Spring or “puquio”	0	1	
River, ditch, lake, lagoon	0	1	
Other	0	2	
Access to sanitary services			
Public sewage system inside the household	8	17	0.731^a^
Cesspool	1	2	
River, ditch, canal or similar	2	3	
Open field or outdoors	0	3	
Other	0	1	
Type of energy for cooking			
Gas	9	26	0.083^a^
Coal	2	0	
Cable TV			
Yes	5	9	0.713^a^
No	6	17	
Internet			
Yes	10	20	0.649^a^
No	1	6

*The questionnaire was not administered to 2 mothers with FCCRE infants and 2 mothers with non-FCCRE infants, ^a^ Fisher’s exact test with bilateral contrast, ^b^ Student’s t-test. SD=standard deviation

## DISCUSSION

Intestinal colonization with AMR bacteria reflects the extent of the spread of bacterial resistance to antimicrobials ^11^. In this study, more than half of the infants were FCCRE (68%), mainly due to *E. coli* (83.3%). The *oqxA* gene was the most frequent (41.4%) among the PMQR genes. In addition, half of the isolates were ESBL producers, and 93.3% of these were carriers of the *bla*_CTX-M_ gene.

In contrast to the high frequency of FCCRE found in this research, Purohit *et al*. reported 12.2% of FCCRE in a sample with children from rural India during the period 2014 - 2016 ^12^. In Peru, Pons *et al*. reported 12.1% of FCCRE in 222 healthy children aged 2 to 12 months, from peri-urban localities of Lima between 2006 - 2007 ^13^.

There are several phenomena that may explain the high frequency of FCCRE. In Peru, the consumption of antibiotics without prescription is frequent. Rojas-Adrianzen *et al*. in 2016, identified that 53.4% of apothecary/pharmacy users incur in this practice ^14^. In addition, environmental contamination with AMR bacteria, mainly due to overcrowding and inadequate excreta management, which are important sources of dissemination of bacterial resistance in developing countries ^15^.

Regarding AMR in other antibiotic families, the high levels of resistance to AMP and SXT stand out with 93.3% and 66.7%, respectively. A study by Kalter *et al*., on 145 healthy children between 3 and 12 months of age, reported AMP and SXT resistance levels of 60% and 57.7%, respectively ^16^. Similarly, Pons *et al*. reported 62.6% and 48.6% resistance to AMP and SXT, respectively ^17^.

Regarding PMQR-associated genes, our results are similar to those reported by Pons *et al.*, who evaluated the frequency of fecal carriers of quinolone-resistant *E. coli* in children younger than 12 months and found *aac(6’)-Ib-cr *and *qnrB* frequencies of 20% and 6%, respectively ^17^. Zhao* et al, *in a 2018 analysis of 736 healthy children aged 3 to 6 years, reported that 8.8% and 1.8% of the 113 ciprofloxacin-resistant isolates carried the *aac(6’)-Ib-cr* and *qnrB* genes, respectively ^18^. In our study, the *oqxA* gene was identified in 41.1% of the isolates. The *oqxA* gene was first reported in Latin America in 2017 by Saba *et al*. in one isolate within 101 cephalosporin-resistant Enterobacteriaceae of clinical origin ^8^.

Fifty percent of the isolates were ESBL producers, which suggests a potential association of resistance transmission between these two antibiotic families, as evidenced in a previous study ^19^. Incidentally, 83.3% of the isolates carrying the *aac(6’)-Ib-cr* gene were also ESBL producers.

Similar to our results, Alcedo *et al*. detected the presence of fecal ESBL-producing *E. coli* in more than 50% of children aged 10 to 20 months, with the *bla*_CTX-M_ gene being the most frequent (98.8%) ^20^. This result is consistent with our findings regarding the predominance of CTX-M-group 1.

On the other hand, ten clonal groups were found in ESBL-producing *E. coli*. Three isolates (15A, 62A, 49A) showed a strong clonal relationship in one of them, even though isolate 15A did not carry the *bla*_CTX-M_ gene. This finding suggests to us that, despite the close phylogenetic relationship, the acquisition of resistance mechanisms has diverse origins ^4^. Furthermore, the high heterogeneity of ESBL-producing *E. coli *reflects a strong influence of the familial environment for the acquisition of these bacteria. This high degree of phylogenetic diversity has been reported by other studies and shows, due to the degree of dissemination, the public health risk that this resistance mechanism represents ^20^.

The economic analysis of the households showed a homogeneous distribution among the groups of infants, which leads us to think of possible conditioning factors that were not evaluated. Likewise, recent studies have shown the relevance of the transmission of AMR bacteria through breast milk to the infant’s intestine, which could be an important factor in the colonization process ^11^. On the other hand, we found that older infants had a higher frequency of being FCCRE, which is related to a higher degree of interaction with the environment, as has been reported by other studies ^13^.

One of the limitations of this study is the limited sample size which may not reflect the overall situation of the studied population. On the other hand, although this study evaluated some genes associated with PMQR, resistance to quinolones mainly results from mutations in the QRDR ^8^. Therefore, it would be important to analyze the other PMQR-associated genes that were not evaluated in this research.

Finally, the studied population showed a high frequency of fecal commensal bacteria resistant to quinolones, partly associated by ESBL, *bla*_CTX-M_ type. This is worrisome because it reflects the extent of the dissemination of AMR bacteria and their possible role in limiting the therapeutic use of antimicrobials in this population. Therefore, these findings alert us to the high presence of AMR bacteria in a population with low direct exposure to antimicrobials, which shows the potential risk of this antibiotic family becoming less useful over time. 
