# Supplementary material for: Identification of multidrug-resistant enterobacteriaceae in fecal samples from infants residing in Talara, Piura, Peru
Source: Rev Peru Med Exp Salud Publica. 2022 Dec 9;39(4):456–62. doi: 10.17843/rpmesp.2022.394.11870 (PMC11397669; doi:10.17843/rpmesp.2022.394.11870)
Supplement: Translation

# Translation: Identificación de enterobacterias multirresistentes a antibióticos en muestras de heces de lactantes residentes en Talara, Piura, Perú

**Authors:** Arturo Octavio Gonzales-Rodríguez, Javier Ignacio Castillo Horna, Edgar Gonzales Escalante

**Affiliations:** 1 Facultad de Medicina Humana, Universidad de Piura, Lima, Perú; 2 Universidad de Buenos Aires, Facultad de Farmacia y Bioquímica, Instituto de Investigaciones en Bacteriología y Virología Molecular (IBaViM), Argentina

**Keywords:** Recién Nacido, *Escherichia coli*, Farmacorresistencia Bacteriana, beta-Lactamasas, Quinolonas, Perú, Coliformes

## Abstract

La colonización fecal en lactantes por bacterias resistentes a los antimicrobianos es un potencial riesgo para futuras terapias antibióticas. Nuestro objetivo fue determinar la frecuencia y características sociodemográficas de lactantes portadores fecales de enterobacterias resistentes a ciprofloxacina (PFRC) y sus genes de resistencia asociados. Analizamos muestras fecales de 41 niños lactantes residentes en el distrito de Talara-Piura, Perú, en 2019. Evaluamos la presencia de 3 genes de resistencia a quinolonas: *aac(6’)-Ib-cr*, *qnrB* y *oqxA* y 2 de betalactamasas: *bla*_CTX-M,_ *bla*_PER-2_.El 68% de lactantes fueron PFRC, *Escherichia coli *(83,3%) fue el más frecuente. El análisis genotípico detectó: *oqxA *(41,1%)*, qnrB *(26,7%) y *aac(6’)-Ib-cr *(20%) y al gen *bla*_CTX-M _en el 93,3% de los aislados con betalactamasas. La elevada frecuencia de PFRC nos alertan sobre el potencial riesgo en la pérdida de utilidad de esta familia antibiótica en el área de estudio.

## INTRODUCCIÓN

La resistencia a los antimicrobianos (RAM) es un serio problema de salud pública. La Organización Mundial de la Salud (OMS) ha estimado que en el 2050 todos los antibióticos serán ineficaces. El principal motivo es la rápida, temprana y amplia diseminación de genes de RAM ^1^. La colonización con bacterias resistentes a los medicamentos es un gran riesgo para la salud, debido a la potencial transferencia de los genes de resistencia a bacterias patógenas y su fácil diseminación entre individuos. Se ha informado que la microbiota intestinal es una de las principales fuentes de infección del tracto urinario, respiratorio y sanguíneo ^2^.

Las quinolonas son una amplia familia de antibióticos, cuya resistencia se incrementó rápidamente en los años noventa ^3^. Las mutaciones en la región determinante de resistencia a quinolonas (QRDR, por sus siglas en inglés) son el principal mecanismo que confiere alto nivel de resistencia. Los marcadores de resistencia a quinolonas mediados por plásmidos (PMQR, por sus siglas en inglés), como son las proteínas Qnr, la enzima AAC(6’)-Ib-cr y la bomba OqxAB, ejercen un bajo nivel de resistencia. Sin embargo, tienen un importante rol en la selección de mutantes cromosómicas en QRDR^ (^ ^3^.

Los ß-lactámicos son la principal familia de antibióticos y las ß-lactamasas de espectro extendido (BLEE) representan el principal mecanismo de resistencia frente a estos antibióticos ^4^. Las enzimas CTX-M, predominantes a nivel mundial, están constituidas por diversos grupos: CTX-M-grupo 1, CTX-M-grupo 2, CTX-M-grupo 8, CTX-M-grupo 9, CTX-M-grupo 25 y grupo KLUC. El grupo 1 y 2 son los de mayor diseminación en América Latina ^4^.

La RAM además de estar relacionada al uso y abuso de los antimicrobianos, también se encuentra relacionada, aunque no directamente, con el nivel socioeconómico familiar y desarrollo económico y sanitario de un país ^5^. Actualmente, es necesario generar evidencia sobre la calidad microbiana del entorno, por ello, el estudio en poblaciones potencialmente protegidas de la colonización por bacterias RAM ofrece dicha oportunidad. Una población de este tipo es la de los niños lactantes, debido a su capacidad motora limitada, escasa exposición a los antimicrobianos y limitada variedad nutricional^ (^ ^6^.

En ese sentido, el objetivo de este estudio fue determinar la frecuencia y características sociodemográficas de lactantes portadores fecales de enterobacterias resistentes a ciprofloxacina (PFRC) y la presencia de genes PMQR y BLEE, en el distrito de Talara, región Piura, Perú.

**MENSAJES CLAVE**

Motivación para realizar el estudio: Los lactantes, debido al consumo nutricional restringido, capacidad motora limitada y escasa exposición antibiótica, son una población protegida de bacterias multidrogoresistentes.

Principales hallazgos: En este estudio, el 68% de los lactantes estuvieron colonizados por bacterias resistentes a quinolonas, siendo E. coli el principal agente causal (83,3%). El principal gen asociado a la resistencia a quinolonas fue oqxA (41,4%,). Asimismo, la mitad de los aislamientos fueron productores de BLEE, y esta resistencia fue causada en el 93,3% de los aislamientos por el gen blaCTX-M.

Implicancias: Estos hallazgos nos alertan sobre la alta presencia de bacterias resistentes a antimicrobianos en una población vulnerable, mostrando el potencial riesgo en la pérdida de utilidad de esta familia antibiótica.

## EL ESTUDIO

Realizamos un estudio descriptivo de corte transversal. Enrolamos a 41 lactantes de entre 3 a 12 meses de edad residentes en el distrito de Talara, región Piura, Perú. De estos, 28 lactantes fueron enrolados de la zona de Talara Baja a partir de información provista por la posta de salud y 13 lactantes de la zona de Talara Alta mediante la identificación en la comunidad de lactantes que cumplieran los criterios de selección.

Las muestras fueron recolectadas entre septiembre y diciembre de 2019. No se realizó un cálculo a priori del tamaño muestral para este estudio. La selección de la muestra fue no probabilística por conveniencia bajo los siguientes criterios de selección: contar con el consentimiento informado por parte de la madre para la participación del menor, haber brindado muestra de heces, tener una frecuencia de amamantamiento mayor a cuatro veces por día y haber nacido por parto por vaginal. Excluimos a lactantes con madres que consumieron antibióticos 15 días previos a la recolección de la muestra fecal.

### Búsqueda e identificación de enterobacterias resistentes a quinolonas

Las muestras de heces fueron transportadas en el medio Cary Blair al laboratorio de Microbiología e Inmunología (LMI) de la Universidad de Piura en Lima para su procesamiento. Las muestras de heces se sembraron en agar MacConkey suplementado con 2 mg/L de ciprofloxacina para el aislamiento presuntivo de enterobacterias resistentes a quinolonas.

La identificación y el perfil de sensibilidad antimicrobiana se realizó con el sistema automatizado Vitek 2 compact (Biomeriux, Francia), la interpretación se realizó siguiendo las recomendaciones del *Clinical and LaboratoryStandards Institute* (CLSI) ^7^. La detección fenotípica de BLEE se realizó mediante el método de doble disco de acuerdo con las recomendaciones del CLSI ^7^.

### Detección de genes de resistencia a quinolonas y betalactámicos

El ADN bacteriano se extrajo usando DNA Purification kit GeneJetGenomic (ThermoScientific), siguiendo las recomendaciones del fabricante. Se determinó la presencia de 3 genes de PMQR: *aac(6’)-Ib-cr*, *qnrB* y *oqxA*, empleando cebadores previamente descritos en la literatura ^8^. Además, entre los aislamientos con evidencia de BLEE se estudió la presencia de dos genes de resistencia asociados a BLEE: *bla*_CTX-M _y*bla*_PER-2_ ^8^. En los aislamientos que se detectó el gen *bla*_CTX-M_, se realizó la búsqueda de los grupos de CTX-M, 1, 2 y 9 ^8^.

### Relación clonal

La relación clonal entre los aislamientos productores de BLEE se determinó mediante la técnica ERIC-PCR, según lo descrito en la literatura ^9^. Se empleó el programa Past, versión 4.0 para integrar, en un dendograma, los resultados a través del algoritmo UPGMA. Se consideró que los aislados que mostraban más del 90% de identidad estaban relacionados clonalmente. 

### Encuesta

Se aplicó un cuestionario estructurado a 37 madres, 11 con hijos no PFRC y 26 con hijos PFRC (no se realizó el cuestionario a 2 madres con hijos PFRC y a 2 madres con hijos sensibles a ciprofloxacina). El cuestionario estuvo basado en las preguntas del capítulo 1 «Características de los Hogares y la población», módulo «Características de las viviendas y los hogares» de la Encuesta Demográfica y de Salud Familiar (ENDES) - 2014^ (^ ^10^. Además, se recolectó información sobre la edad y sexo de los lactantes.

### Análisis estadístico

El análisis estadístico se realizó mediante el programa SPSS Statistics for Windows, Version 25.0. Armonk, NY: IBM Corp. La descripción de las variables cualitativas se realizó a través de gráficas de frecuencia y las variables cuantitativas en tablas. Se aplicó la prueba exacta de Fisher para evaluar diferencias entre los grupos portadores de enterobacterias resistentes y sensibles a quinolonas y la prueba t-Student para la comparación de medias. Los valores p <0,05 se consideraron significativos. 

### Consideraciones éticas

La aprobación ética del estudio principal «Asociación del estrés postnatal frente a la calidad del microbioma de la leche materna y su relación con la anemia ferropénica en niños lactantes» (Código del proyecto: PI2008-UDEP) fue otorgada por el Comité Institucional de Ética en Investigación de la Facultad de Medicina Humana de la Universidad San Martín de Porras. El consentimiento y asentimiento informado fueron obtenidos de las madres de cada lactante previo al enrolamiento. 

## HALLAZGOS

Se enrolaron a 41 lactantes, con un promedio de edad de 7,5 meses, siendo el 48,6% hombres y el 51,4% mujeres. El 68,3% (28/41) de los lactantes eran PFRC. 

*Pruebas de identificación bacteriana y susceptibilidad antibiótica*

Se recuperaron 30 aislamientos resistentes a ciprofloxacino, provenientes de 28 lactantes (en dos lactantes se recuperaron dos tipos de bacterias). Las especies aisladas fueron *Escherichia coli* (25/30), *Citrobacter freundii (*2/30), *Enterobacter cloacae *subsp *cloacae *(1/30), *Klebsiella pneumoniae *subsp *pneumoniae*(1/30) y *Hafnia paralvei *(1/30). El perfil de sensibilidad a los antimicrobianos se muestra en la Figura 1, resaltando los niveles de resistencia a ampicilina (AMP) y trimetoprim/sulfametoxazol (SXT), 93,3% y 66,7%, respectivamente. Además, la evaluación fenotípica de BLEE, permitió detectarlo en 15 de 30 aislamientos.

**Figura 1:** Perfil de susceptibilidad antimicrobiana (n =30). AMP: ampicilin; TZP: piperacilina/tazobactam; CFZ: cefzazolina; CAZ: ceftazidima; CRO: ceftriaxona; FEP: cefepime; ERT: ertapenem; IPM: imipenem; AMK: amikacina; GM: gentamicina; TOB: trobamicina; LVX: levofloxacina; NIT: nitrofurantoina y SXT: trimetoprim/sulfametoxazol.

*Detección genotípica y perfil de clonalidad*

Se detectaron los siguientes genes de PMQR: *oqxA* (13/30), *qnrB* (8/30) y *aac(6’)-Ib-cr *(6/30). En los productores de BLEE se detectó el gen *bla*_CTX-M _(14/15). Se determinó que las cepas con el gen *bla*_CTX-M_ pertenecían a los siguientes grupos: *bla*_CTX-M-grupo1_ (12/14), *bla*_CTX-M-grupo 2 _(4/14) y *bla*_CTX-M-grupo 9_ (4/14). No se detectó la presencia del gen *bla*_PER-2_ (Figura 2).

**Figura 2:** Distribución de genes de resistencia tipo BLEE y PMQR según fenotipo y género bacteriano. , : Presencia; : Ausencia.

El estudio de la relación filogenética entre los aislamientos de *E. coli *productores de BLEE, evidenció hasta 10 grupos clonales; los aislamientos relacionados fueron: 15A, 62A y 49A (Figura 3).

**Figura 3:** . Dendograma basado en la distancia genética euclidiana usan-do el método de análisis no apareado en la media aritmética (UPGMA), construido mediante análisis de presencia y ausencia de las regiones inter-genéticas consenso por PCR (ERIC-PCR), para los aislamientos de E. coli productores de BLEE.

*Análisis demográfico y económico*

No se evidenció diferencia estadística entre los grupos de lactantes portadores fecales de enterobacterias sensibles y resistentes a ciprofloxacina con respecto al sexo. Sin embargo, sí se pudo observar diferencia estadística según la edad (p = 0,007). En la Tabla 1 se muestran los resultados para otras variables del hogar. Finalmente, no se encontró diferencia estadística entre nivel económico y alguna de las variables evaluadas.

**Tabla 1:** Análisis sociodemográfico entre grupo de lactantes portadores fecales con enterobacterias resistentes a ciprofloxacina (PFRC) y lactantes no PFRC.

Variables	Lactantes no PFRC (n =11)*	Lactantes PFRC (n = 26)*	Valor de p
Edad (meses)- media (DE)	6,27 (2,49)	8,64 (2,18)	0,007^b^
Sexo			
Hombre	6	12	0,721^a^
Mujer	5	14	
Tipo de vivienda			
Casa independiente	8	14	0,609^a^
Departamento en edificio	0	1	
Vivienda en quinta	0	1	
Vivienda en casa de vecindad (callejón, solar o corralón)	0	0	
Choza o cabaña	1	7	
Vivienda improvisada	2	3	
Material del piso de la vivienda			
Parqué o madera pulida	1	1	0,862^a^
Láminas asfálticas, vinílicos o similares	0	2	
Losetas, terrazos o similares	2	4	
Cemento	7	17	
Tierra	1	2	
N° habitaciones/N° hab dormir, media (DE)	1,01 (0,77)	1,10 (1,54)	0,813^b^
Condición vivienda			
Alquilada	0	4	0,538^a^
Propia, totalmente pagada	5	8	
Propia, por invasión	3	7	
Otra	3	7	
Acceso al agua			
Red pública, dentro de la vivienda	9	18	0,863^a^
Red pública, fuera de la vivienda, pero dentro de edificio	0	0	
Pilón o pileta de uso público	1	2	
Camión-cisterna u otro similar	1	2	
Pozo (agua subterránea)	0	0	
Manantial o puquio	0	1	
Río, acequia, lago, laguna	0	1	
Otro	0	2	
Acceso a servicios higiénicos			
Red pública de desagüe dentro de la vivienda	8	17	0,731^a^
Pozo ciego o negro	1	2	
Río, acequia, canal o similar	2	3	
Campo abierto o al aire libre	0	3	
Otros	0	1	
Tipo de energía para cocinar			
Gas	9	26	0,083^a^
Carbón	2	0	
Televisión por cable			
Presenta	5	9	0,713^a^
No presenta	6	17	
Internet			
Presenta	10	20	0,649^a^
No presenta	1	6	

*No se realizó el cuestionario a 2 madres con hijos PFRC y a 2 madres con hijos no PFRC, ^a^ Prueba exacta de Fisher con contraste bilateral,^b^ Prueba t-Student. DE=desviación estándar

## DISCUSIÓN

La colonización intestinal con bacterias con RAM es un reflejo del alcance de la diseminación de la resistencia bacteriana a los antimicrobianos ^11^. En este estudio, más de la mitad de los lactantes fueron PFRC (68%), principalmente debido a *E. coli *(83,3%). El gen *oqxA* fue el más frecuente (41,4%) entre los genes PMQR analizados. Además, la mitad de los aislamientos fueron productores de BLEE, el 93,3% de estos fueron portadores del gen *bla*_CTX-M._

En contraste con la alta frecuencia de PFRC encontradas en esta investigación, Purohit *et al*. reportó un 12,2% de PFRC en una muestra con niños provenientes de la zona rural de India en el periodo 2014 - 2016 ^12^. En Perú, Pons *et al.*, reportó 12,1% de PFRC en 222 niños sanos de entre 2 a 12 meses, de localidades periurbanas de Lima entre 2006 - 2007 ^13^. 

Existen diversos fenómenos que pueden explicar la elevada frecuencia de PFRC. En el Perú, el consumo de antibióticos sin receta médica es frecuente. Rojas-Adrianzen *et al*. en 2016, identificaron que el 53,4% de usuarios de boticas/farmacias incurre en esta práctica ^14^. Además, la contaminación ambiental con bacterias RAM, principalmente debido al hacinamiento y al manejo inadecuado de las excretas, son fuentes importantes de diseminación de la resistencia bacteriana en países en vías de desarrollo ^15^.

Respecto a la RAM en otras familias, resaltan los altos niveles de resistencia a AMP y SXT, 93,3% y 66,7%, respectivamente. Kalter *et al. *en un estudio con 145 niños sanos de entre 3 y 12 meses de edad, detectaron niveles de resistencia a AMP y SXT del 60% y 57,7%, respectivamente ^16^. De igual forma, Pons *et al.* reportó 62,6% y 48,6% de resistencia a AMP y SXT, respectivamente ^17^.

Con relación a los genes asociados a PMQR, nuestros resultados son similares a lo reportado por Pons *et al*, quienes evaluaron la frecuencia de portadores fecales de *E. coli* resistentes a quinolonas en niños menores de 12 meses y encontraron frecuencias de *aac(6’)-Ib-cr *y *qnrB *del 20% y 6%, respectivamente ^17^. Mientras que, Zhao *et al*, en un análisis realizado en el 2018, con 736 niños sanos de entre 3 a 6 años, detectaron que en 113 aislamientos resistentes a ciprofloxacina el 8,8% y 1,8% eran portadores de los genes *aac(6’)-Ib-cr *y *qnrB*, respectivamente ^18^. En este estudio, el gen *oqxA *fue identificado en el 41,1% de los aislados. El gen *oqxA* fue reportado por primera vez en América Latina en 2017 ^8^; Saba *et al*. lo identificaron en un aislamiento dentro de 101 enterobacterias de origen clínico resistentes a cefalosporinas ^8^. 

Con respecto a la resistencia a betalactámicos, el 50% de los aislamientos fueron productores de BLEE, sugiriendo una potencial asociación de la transmisión de la resistencia en estas dos familias antibióticas, como ha sido evidenciada en otro estudio ^19^. A propósito, el 83,3% de los aislamientos portadores del gen *aac(6’)-Ib-cr* también fueron productores de BLEE.

Similar a nuestros resultados, Alcedo *et al.* detectaron, en más del 50% de niños de entre 10 a 20 meses, la presencia fecal *E. coli *productoras de BLEE, siendo el gen *bla*_CTX-M _el de mayor frecuencia (98,8%) ^20^. Este resultado es consistente con nuestros hallazgos con relación al predominio del CTX-M-grupo 1. 

Por otro lado, se observaron diez grupos clonales en las *E. coli *productoras de BLEE. En uno de ellos, tres aislamientos (15A, 62A, 49A) presentaron una alta relación clonal, a pesar de que el aislado 15A no era portador del gen *bla*_CTX-M_. Este hallazgo nos sugiere que, a pesar de la estrecha relación filogenética, la adquisición de mecanismos de resistencia tiene diversos orígenes ^4^. Además, la alta heterogeneidad de *E. coli* productoras de BLEE refleja una fuerte influencia del ambiente familiar para la adquisición de estas bacterias. Este elevado grado de diversidad filogenética ha sido observado en otros estudios y manifiesta, por el grado de diseminación, el riesgo en la salud pública que presenta este mecanismo de resistencia^ (^ ^20^.

El análisis económico de las viviendas evidenció una distribución homogénea entre los grupos de lactantes, lo cual nos hace pensar en factores condicionantes no evaluados. Asimismo, recientes estudios han manifestado la relevancia en la transmisión de bacterias RAM a través de la leche materna al intestino del lactante, con lo cual este podría constituir un factor importante en el proceso de colonización ^11^. Por otro lado, se pudo constatar que los lactantes con mayor edad tuvieron mayor frecuencia de PFRC, lo cual se encuentra relacionado con un mayor grado de interacción con su ambiente, como ha sido observado en otros estudios ^13^.

Es importante resaltar que este estudio presenta limitaciones debido al tamaño muestral, el cual puede no reflejar la situación global de la población estudiada. Por otro lado, aunque este estudio evaluó algunos genes asociados a PMQR, la resistencia a quinolonas principalmente surge, como efecto de mutaciones en la QRDR ^8^. Por lo que sería importante analizar otros genes asociados a PMQR no evaluados en esta investigación.

Finalmente, la población estudiada presentó una alta frecuencia de bacterias comensales fecales resistentes a quinolonas, en parte acompañadas por BLEE, tipo *bla*_CTX-M_. Esto es preocupante porque refleja la extensión de la diseminación de bacterias RAM y su eventual rol en la limitación del uso terapéutico de los antimicrobianos en esta población. Por lo cual, estos hallazgos nos alertan sobre la alta presencia de bacterias RAM en una población con baja exposición directa a los antimicrobianos, mostrando el potencial riesgo en la pérdida de utilidad de esta familia antibiótica en el área de estudio.
